# Starting Solo: Experiences and Challenges of Early Career Psychiatrists in Private Practice in India

**DOI:** 10.7759/cureus.89101

**Published:** 2025-07-30

**Authors:** Ashvin Chouhan, Asish A Choudhury, Simran Sandhu

**Affiliations:** 1 Psychiatry, Mahatma Gandhi Memorial Medical College, Indore, IND; 2 Psychiatry, Institute of Medical Sciences and SUM Hospital, Bhubaneswar, IND

**Keywords:** early career psychiatrists, india, mental health, mentorship, private practice, professional challenges, psychiatry, stigma

## Abstract

Background

As India faces a severe shortage of psychiatrists, a growing number of early career psychiatrists are opting for private practice. However, limited literature explores their experiences, challenges, and preparedness in this setting.

Objective

The aim of this study was to assess the motivations, expectations, current practices, challenges, and psychological impact among young Indian psychiatrists in early private practice.

Methods

A cross-sectional online survey was conducted among 100 early career psychiatrists engaged in private practice across India. A structured, self-designed, and pilot-tested questionnaire was disseminated using convenience and snowball sampling. Quantitative data were analyzed using descriptive statistics.

Results

Most respondents were aged 31-35 years, had less than four years of post-PG (postgraduate) experience, and practiced solo in urban or semi-urban areas. The primary motivation for entering private practice was independence (47%), followed by work-life balance (29%). Key challenges included financial instability (53%), limited patient inflow (46% saw zero to two patients per day), and lack of training in non-clinical skills. In terms of insurance, only 36% had indemnity insurance. Furthermore, nearly one-third (32%) found private practice more difficult than expected, citing unpreparedness in marketing, administration, and patient communication. While 57% reported positive psychological impact, 20% experienced mental health difficulties. Feelings of isolation were common (63%). Engagement with professional bodies was high, but perceived usefulness was low. High perceived stigma and generational differences in help-seeking were also noted.

Conclusion

Early private practice in psychiatry poses significant systemic, administrative, and emotional challenges. There is a pressing need for curricular reforms, mentorship opportunities, and policy-level support to facilitate sustainable and satisfying private practice pathways for young psychiatrists in India.

## Introduction

Private medical practice has long been an integral part of healthcare delivery in India, serving as a primary point of contact for a large section of the population. In the field of psychiatry, private practice plays a particularly important role, as access to mental health services within public healthcare systems remains limited and overburdened [1].

Private practitioners in India, especially in mental health, work in a system with very little support and few clear rules. A study from Maharashtra showed that different authorities set different rules for private doctors, which creates confusion and increases stress for practitioners [2]. Another major problem is the severe shortage of psychiatrists - India has only around 0.3 psychiatrists per 100,000 people, far below what is needed [3].

In India, many young psychiatrists are choosing to start their own private practice soon after completing their postgraduate (PG) training. While this allows more freedom and independence, it also brings several challenges that are often not discussed enough. A survey of 86 private practitioners demonstrated that undergraduate curricula poorly prepare providers for mental health issues in primary care settings [4]. Additionally, private practitioners often face pressures related to the commercialization of healthcare, such as profit-driven expectations and corporate influence [5]. These factors can sometimes clash with clinical judgment and compromise the psychiatrist’s professional autonomy.

Despite the growing number of young psychiatrists entering private practice in India, there is limited research exploring their lived experiences, motivations, and challenges in this setting. Much of the existing literature focuses on public sector psychiatry or broader healthcare delivery, overlooking the unique stressors associated with setting up and sustaining a solo practice, such as lack of mentorship, administrative burdens, professional isolation, and the stigma still attached to mental health. This study aims to fill this gap by capturing the perspectives of young Indian psychiatrists currently engaged in private practice.

## Materials and methods

A cross-sectional, online survey-based study was conducted to understand the experiences, expectations, and challenges faced by private psychiatry practitioners in India. A structured, self-designed questionnaire was developed by the research team after reviewing relevant literature and engaging in informal discussions with early-career psychiatrists [5-8].

Content validity was established by a panel of three senior psychiatrists with over 10 years of experience in clinical and academic psychiatry, who reviewed each item for relevance, clarity, and domain representation. Face validity was assessed informally by sharing the draft tool with 10 early career psychiatrists not involved in the study design. Feedback was obtained to ensure that the questions were easily understandable, contextually appropriate, and relevant to clinical practice. Although pilot testing was conducted, formal reliability testing (e.g., internal consistency via Cronbach’s alpha) was not performed due to the exploratory nature of the study. Feedback obtained from the pilot group was used to make minor revisions.

The questionnaire comprised questions about demographics, pre-practice expectations and preparation, current practice and challenges, expectations versus reality, emotional and psychological impact, associations and networks, and stigma perception. Questions were of different formats, including single-choice, multiple-choice, and Likert scale responses (Table 1).

**Table 1:** Survey questionnaire for early career psychiatrists in private practice PG, postgraduate

Section	Question
Demographic information	Name (optional)
	City/area of practice
	Age
	Gender
	Post-PG years of experience in psychiatry
	What degree have you obtained?
	Type of practice
	Setting of practice
Pre-practice expectations and preparation	What inspired you to pursue psychiatry as a specialty?
	What were your primary motivations for starting a private practice?
	What was your biggest fear or concern before starting your practice?
	How did you prepare yourself before starting your practice?
Current practice and challenges	Did you feel any need for receiving a formal training or mentorship on running a private practice?
	How long have you been running your private practice?
	How would you describe your current patient inflow?
	What are the most common mental health issues you encounter in your practice?
	On average, how much time do you spend with a new patient during the first consultation?
	What is the usual consultation fee for a first-time psychiatric visit (in INR)?
	What is the usual consultation fee for a follow-up psychiatric visit (in INR)?
	Does your fee vary if the time of consult extends?
	Do you personally conduct psychotherapy or counselling sessions?
	What proportion of your consultations are via teleconsultation?
	How many days do you offer teleconsultation in a week?
	What is the arrangement for medicines in your clinic?
	Marketing of your practice depends on what factors?
	Do you currently use any practice management software?
Expectations vs reality	Do you find local listings or lead generation websites useful?
	Have you faced any legal or regulatory issues since starting your practice?
	Have you taken a professional indemnity insurance?
Emotional and psychological impact	How satisfied are you with your current practice?
	How does your current experience compare to your initial expectations?
	What aspects of running a private practice were you unprepared for?
Associations and networks	What has been the most rewarding aspect of your private practice?
	How has private practice affected your mental health?
	Do you feel isolated or unsupported in your practice?
Societal and cultural context	What coping mechanisms do you use to manage stress or burnout?
	Have you joined any professional associations or networks?
	What associations have you joined?
	How helpful have they been?

The survey was disseminated online using convenience and snowball sampling methods. The questionnaire was shared via Google Forms through professional WhatsApp groups, Facebook, Instagram, and other relevant social media platforms targeting psychiatrists. Participation was entirely voluntary and anonymous. No financial or material incentives were provided. Only qualified early career private psychiatrists (defined as within the first five years of independent post-qualification private practice) currently practicing in India were eligible to participate. Both full-time and part-time private practitioners were included. To ensure eligibility, the first section of the survey required participants to self-declare their qualification and current private practice status before proceeding to the rest of the questionnaire. Responses from individuals who did not confirm these criteria were excluded. The study was of one-month duration, starting from June 1, 2025, to July 1, 2025.

The sample size for this cross-sectional survey was calculated using the formula for estimating a proportion, assuming a 95% confidence level (Z = 1.96), an expected proportion (p) of 0.5 to maximize sample size in the absence of prior data, and a margin of error (d) of 10%. Based on this, the minimum required sample size was calculated to be 96 participants. A total of 100 participants filled the survey.

Ethical approval was obtained from the institutional ethics committee prior to study initiation, with approval obtained on May 1, 2025 (MGM/EC/May/9).

Descriptive statistics were applied, and analysis was conducted using SPSS Version 25 (IBM Corp., Armonk, NY).

## Results

Sociodemographic data

The study found that a significant proportion of respondents were between 31 and 35 years of age (60%), with a relatively balanced gender distribution. Regarding years of post-PG experience, 78% had less than four years of experience. A majority (80%) held an MD in Psychiatry, while 13% held a DNB and 7% held a DPM. Most respondents (80%) were engaged in solo practice, operating primarily out of their own or rented chambers (82%), with the rest affiliated with corporate setups or rehabilitation centers (18%). In terms of location, urban areas accounted for 70% of practices, semi-urban accounted for 28%, and only 2% were based in rural areas (Table 2).​​​​

**Table 2:** Sociodemographic profile of 100 participants PG, postgraduate; MD, doctor of medicine; DNB, diplomate of national board; DPM, doctor of podiatric medicine

Sociodemographic variable	N	%
Region of practice in India		
Central	29	29
Western	20	20
Eastern	19	19
Northern	18	18
Southern	14	14
Age (years)		
25-30	21	21
31-35	60	60
36-40	17	17
>40	2	2
Gender		
Male	56	56
Female	44	44
Post-PG years of experience		
<1 year	6	6
1–2 years	30	30
3–4 years	42	42
≥5 years	22	22
Degree obtained		
MD psychiatry	80	80
DNB psychiatry	13	13
DPM	7	7
Type of practice		
Solo	80	80
Group/partnership	20	20
Setting of practice		
Own chamber/rented	82	82
Corporate/rehabilitation center	18	18
Location of practice		
Urban	70	70
Semi urban	28	28
Rural	2	2

Pre-practice expectations and motivations

The majority of psychiatrists (78%) reported that their decision to pursue psychiatry as a specialty stemmed from a personal interest in mental health. Other less common motivations included choosing psychiatry by chance or default (16%), a desire to address the mental health gap in India (4%), and influence from mentors or role models (2%).

When asked about their primary motivation for entering private practice, nearly half (47%) cited the desire for independence and autonomy, followed by the pursuit of work-life balance (29%), the intention to provide personalized care (13%), dissatisfaction with institutional setups (9%), and lack of government job opportunities (2%).

Financial instability (53%) was the most frequently cited concern before starting their private practice, followed by worries about lack of patient inflow (29%), competition from established practitioners (9%), stigma around mental health in the local area (6%), and difficulties in managing administrative responsibilities (3%).

When asked about preparatory steps taken before beginning private practice, 51% reported conducting research into the local mental health landscape. However, only 29% had actively sought mentorship or guidance, and an even smaller proportion engaged in financial planning (16%) or networking with other healthcare providers (4%). Importantly, 55% expressed the need for formal training or mentorship on running a private psychiatric practice (Table 3).

**Table 3:** Pre-practice expectations and motivations (N=100)

Variable	n			%
Inspiration to pursue psychiatry as a specialty				
Personal interest in mental health	78			78
Preferred by chance, not by choice	16			16
Desire to address mental health gap in India	4			4
Influence of mentors or role models	2			2
Primary motivation for starting a private practice				
Desire for independence/autonomy	47			47
Work-life balance/flexibility	29			29
Desire to provide personalized care	13			13
Dissatisfaction with institutional settings	9			9
Lack of government job opportunities	2			2
Concerns before starting private practice				
Financial instability	53			53
Lack of patient inflow	29			29
Competition from established practitioners	9			9
Stigma around mental health in the area	6			6
Managing administrative tasks	3			3
Preparation before starting private practice				
Researching local mental health landscape		51		51
Seeking mentorship/guidance		29		29
Financial planning		16		16
Networking with other healthcare providers		4		4
Need for receiving formal training or mentorship on running a private practice				
Yes		55		55
No		45		45

Current practice and challenges

The majority of participants had relatively recent experience in private practice, with 26% practicing for three to five years, 33% for one to three years, 22% for six months to one year, and 19% for less than six months. When asked about patient inflow, nearly half (46%) reported seeing 0-2 patients per day, followed by 3-5 patients (23%), 5-10 patients (21%), and more than 10 patients daily (10%).

Anxiety disorders (35%) and mood disorders (30%) were the most common mental health issues encountered, followed by substance use disorders (10%), sleep disorders (8%), child and adolescent mental health issues (5%), and sexual disorders (4%). A small number (8%) reported encountering other types of cases.

In terms of time spent with new patients during the initial consultation, 53% reported spending more than 20 minutes, 45% spent 10-20 minutes, and only 2% spent less than 10 minutes. The majority of respondents charged a consultation fee between ₹400-700, both for first-time visits (59%) and follow-ups (59%). A smaller proportion charged less than ₹400 (17% first-time; 27% follow-up), and only 10% (first-time) and 8% (follow-up) charged more than ₹1,000. Notably, 77% of psychiatrists did not vary their fee even if the consultation extended beyond the usual time.

When it came to psychotherapy, 65% reported conducting it occasionally, while 21% did so regularly, and 14% referred patients to psychologists instead. Tele-consultation was integrated into the practices of most respondents: 62% conducted 1-25% of their consultations online, 20% offered tele-consults daily or three to five days a week, while 18% did not offer it at all.

For medication-related logistics, 47% had tie-ups with nearby pharmacies, 25% advised patients to purchase medicines externally, 16% maintained an in-clinic stock, and 12% operated an in-house pharmacy.

Marketing of services primarily relied on word-of-mouth (60%), with fewer using social media platforms (18%), collaboration with other providers (10%), online directories (7%), or community outreach (5%). Regarding digital practice tools, 40% were considering using practice management software, 14% used it regularly, and 18% were unfamiliar with it altogether. In terms of online visibility, only 28% found lead-generation platforms such as Practo or Justdial useful, while 40% did not find them helpful, and 32% were unsure.

Only 36% had professional indemnity insurance, and 8% reported facing legal or regulatory issues since starting practice (Table 4).

**Table 4:** Current practice and challenges (N=100)

Variable	Response	n	%
How long have you been running your private practice?	1 year to 3 years	33	33
	3 to 5 years	26	26
	6 months to 1 year	22	22
	Less than 6 months	19	19
How would you describe your current patient inflow?	0-2/day	46	46
	>10/day	20	20
	3-5/day	18	18
	5-10/day	16	16
What are the most common mental health issues you encounter in your practice?	Anxiety disorders	35	35
	Mood disorders (depression, mania etc.)	30	30
	Substance use disorders (alcohol, opioid use disorders etc.)	10	10
	Sleep disorders	8	8
	Sexual disorders	4	4
	Child and adolescent mental health issues	5	5
	Other	8	8
On average, how much time do you spend with a new patient during the first consultation?	>20 minutes	53	53
	10- 20 minutes	45	45
	<10 minutes	2	2
What is the usual consultation fee for a first-time psychiatric visit (in INR)?	400-700	59	59
	<400	17	17
	700-1,000	14	14
	>1,000	10	10
What is the usual consultation fee for a follow up psychiatric visit (in INR)?	400-700	59	59
	<400	27	27
	>1,000	8	8
	700-1,000	6	6
Does your fee vary if the time of consult extends?	No	77	77
	Yes	23	23
Do you personally conduct psychotherapy or counselling sessions as part of your private practice?	Occasionally	65	65
	Yes, regularly	21	21
	No, I refer patients to a psychologist	14	14
Approximately what proportion of your psychiatric consultations are conducted via tele-consultation (online/video/phone)?	1-25%	73	73
	0%	14	14
	>75%	6	6
	51-75%	5	5
	26-50%	2	2
In a typical week, how many days do you offer tele-consultations?	1 -2 days	38	38
	0 days	24	24
	Everyday	21	21
	3-5 days	17	17
What is the arrangement for medicines in your clinic?	Tie up with a nearby pharmacy for patient convenience	47	47
	Patients buy medicines from outside	25	25
	In-clinic stock of commonly prescribed medications	16	16
	In-house pharmacy run separately	12	12
The marketing of your practice largely depending upon what factors?	Word of mouth	60	60
	Collaboration with other healthcare providers	10	10
	Social media platforms	18	18
	Online directories such as Justdial and Practo	7	7
	Workshops or community outreach programs	5	5
Do you currently use any practice management software in your clinic?	No, but I am considering it	40	40
	I am not familiar with such software	18	18
	Occasionally	15	15
	Yes, regularly	14	14
	No, and I do not intend to use one	13	13
Do you find local listings or lead generation websites (e.g., Justdial, Practo, Sulekha) useful for your private practice?	No	40	40
	Maybe	32	32
	Yes	28	28
Have you faced any legal or regulatory issues since starting your practice?	No	92	92
	Yes	8	8
Have you taken a professional indemnity insurance in your private practice?	No	64	64
	Yes	36	36
How satisfied are you with your current practice?	Neutral	42	42
	Satisfied	33	33
	Very satisfied	11	11
	Dissatisfied	10	10
	Very dissatisfied	4	4

Expectations versus reality

**Figure 1:**
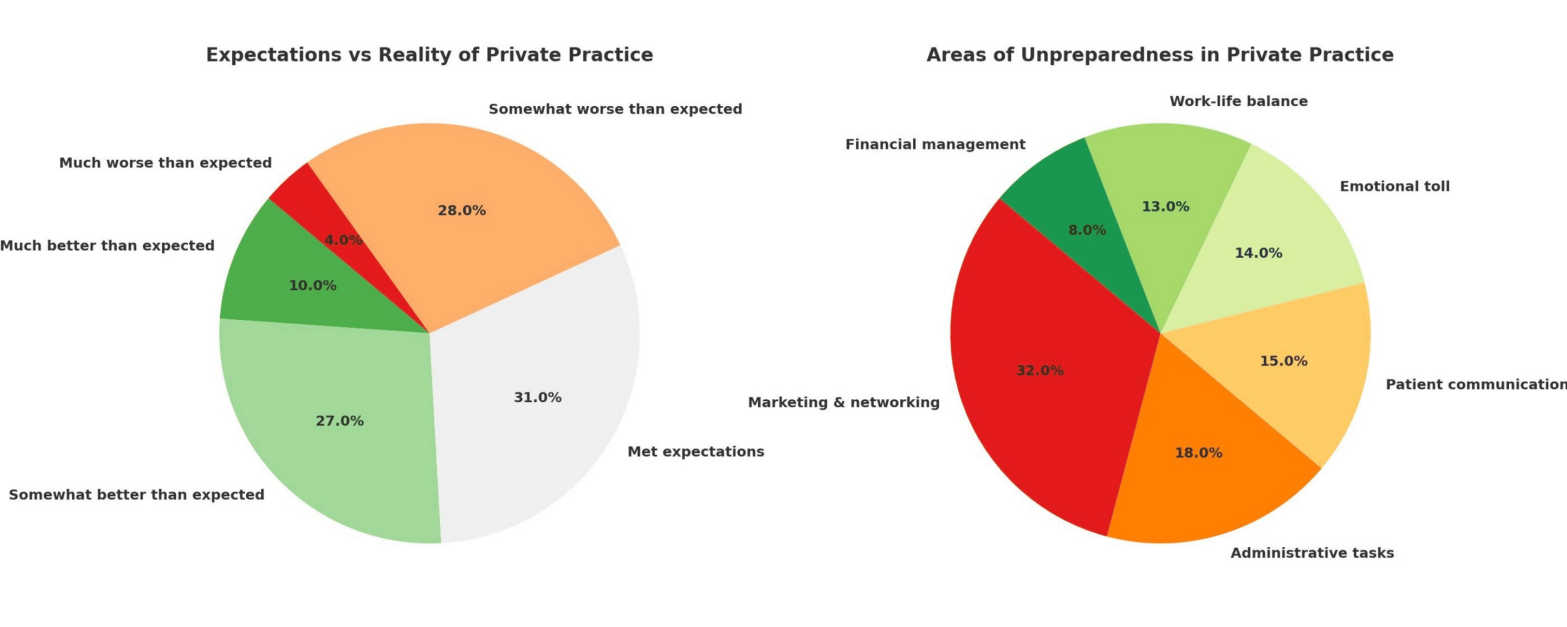
Key experiences reported by early- career psychiatrists in private practice (N=100)

A significant proportion of early career psychiatrists reported that private practice did not meet their expectations. Specifically, 28% stated their experience was somewhat worse than expected, while an additional 4% felt it was much worse than expected, totaling 32% who found the reality to be more challenging than anticipated. Regarding aspects for which they felt unprepared, the most commonly cited area was marketing and networking (32%), followed by administrative tasks (18%), patient communication (15%), emotional burden (14%), work-life balance (13%), and financial management (8%) (Figure 1). Despite these challenges, participants also highlighted several rewarding elements of private practice. The most frequently mentioned was positive patient outcomes (44%), followed by work flexibility (23%), professional autonomy (15%), and financial independence (15%), and a smaller proportion (3%) found fulfilment in building trust within the community.

Psychological impact and networking

**Figure 2:**
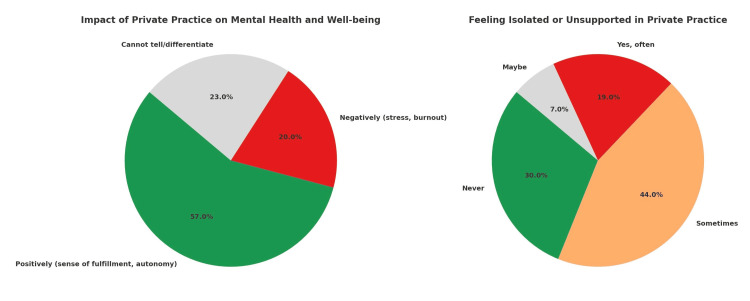
Mental health impact and emotional experience in private practice (N=100)

While 57% reported a positive effect, citing increased autonomy and a sense of fulfilment, 20% stated that private practice had negatively impacted their mental health, primarily due to stress and burnout. Another 23% were uncertain or unable to differentiate the psychological impact, suggesting that the effects were subtle or evolving over time. When asked about feelings of isolation or lack of support, 44% said they sometimes felt isolated, 19% reported often feeling unsupported, 30% had never experienced such isolation, and 7% were unsure (Figure 2). In response to these challenges, psychiatrists most commonly coped by seeking support from family and friends (47%) and engaging in hobbies or leisure activities (34%). Fewer relied on professional avenues such as therapy (6%) or peer support groups (13%). Notably, while many were affiliated with professional bodies - primarily the Indian Psychiatric Society (IPS) - the perceived helpfulness of these associations varied considerably (Table 5), suggesting that such memberships may not always translate into felt support during early private practice.

**Table 5:** Engagement with professional bodies and their perceived utility in private psychiatric practice (N=100) IPS, Indian Psychiatric Society; IMA, Indian Medical Association

Variable	Response	n	%
Joined any professional associations	Yes	76	76
	No	24	24
Professional associations joined	Only IPS	46	46
	Both IPS and IMA	22	22
	None	24	24
	Only IMA	4	4
	Other	4	4
Helpfulness of associations (1=low, 5=high)	1	32	32
	3	32	32
	2	27	27
	4	5	5
	5	4	4

Perceived stigma in society

**Figure 3:**
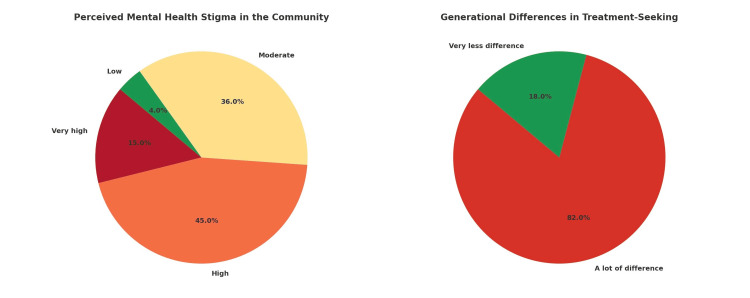
Perceived mental health stigma and generational differences in treatment-seeking (N=100)

Overall, 45% of respondents rated the level of perceived stigma in their community as high, 15% rated it as very high, and 36% considered it moderate. Only 4% felt that stigma was low. When asked about generational trends in treatment-seeking, a resounding 82% of respondents observed significant generational differences, with younger individuals more likely to approach psychiatrists for help, while 18% perceived little to no difference (Figure 3). The consequences of this stigma were evident in clinical interactions. The most reported effects included reluctance to seek help initially (37%), discouragement from family members (30%), poor treatment follow-up (22%), and treatment delays (6%). In response, 45% of practitioners used social media to raise awareness, 35% conducted workshops or public programs, and 18% collaborated with schools or colleges to address stigma at the grassroots level.

## Discussion

This study provides a comprehensive overview of the challenges encountered by early career psychiatrists embarking on private practice in India. The findings highlight significant gaps in preparedness, emotional toll, systemic barriers, and sociocultural factors that shape the practice landscape for young mental health professionals.

The study found that when it came to starting private practice, desire for independence and autonomy (47%) emerged as the leading driver, followed by work-life balance (29%) and the wish to provide personalized care (13%). These findings align with existing research suggesting that private practice is increasingly seen as a pathway to flexible, patient-centered care [7,8]. The desire for independence also likely stems from dissatisfaction with institutional environments and the limited opportunities available in the public sector, which continues to suffer from workforce shortages and systemic constraints [3,9].

Interestingly, only a small proportion (16%) reported seeking formal mentorship, and even fewer engaged in financial or logistical planning prior to starting their practice. This underscores a significant preparatory gap among new practitioners - a finding echoed in earlier studies [5]. The fact that over half (55%) of participants expressed a need for formal training or mentorship programs reinforces the urgency of curricular reform to better prepare trainees for private sector transitions.

The findings reflect a majority having less than three years of experience and a relatively low daily patient inflow (zero to five patients for 69%). This is in line with previous literature suggesting that young psychiatrists often face initial difficulties in building patient trust and visibility in private settings [10]. Despite these challenges, a commendable proportion of respondents (53%) reported spending over 20 minutes with new patients, suggesting a patient-centered approach uncommon in high-volume clinical environments [11].

Anxiety and mood disorders emerged as the most frequently encountered conditions, consistent with epidemiological data showing these disorders as the most prevalent in outpatient psychiatric settings [1].

Tele-consultation had been adopted by over 80% of respondents to some degree, reflecting a broader trend toward digitalization accelerated by the COVID-19 pandemic [12,13]. However, only 14% reported using digital practice management software regularly, and a considerable proportion (18%) were unaware of such tools, highlighting an underutilization of technology that could ease administrative burden and enhance efficiency.

Marketing and visibility were reliant on word of mouth (60%), with social media (18%) and networking with other providers (10%) playing secondary roles. These figures suggest limited structured marketing efforts, possibly due to ethical concerns or lack of formal training in practice promotion. This is supported by the finding that 32% of participants felt most unprepared for marketing and networking activities.

The low uptake of professional indemnity insurance (only 36%) and the presence of legal or regulatory issues in 8% underscore gaps in medicolegal awareness. Prior studies have also highlighted that private psychiatric practitioners often operate without robust legal safeguards, increasing their vulnerability in high-stakes clinical situations [8].

Despite entering private practice with aspirations for autonomy, flexibility, and meaningful patient care, nearly one-third of respondents reported that their experience was worse than anticipated (Figure 1). This gap between expectation and lived experience has been previously noted in literature examining early career transitions in medicine, where idealism often confronts systemic and operational realities [14]. In psychiatry, this mismatch may be exacerbated by the emotional labor, stigma-related barriers, and lack of institutional support typical of solo private practice - challenges that are particularly pronounced in communities where myths and negative perceptions about mental illness persist [15,16].

Respondents felt particularly unprepared for non-clinical aspects of practice. Marketing and networking emerged as the biggest challenges (32%), followed by administrative work, patient communication, and financial management. These findings reflect a consistent theme in Indian psychiatric training - its strong clinical foundation but limited focus on business, communication, or leadership skills [17]. This lack of preparation often leads to high levels of stress and disillusionment early in the professional journey.

At present, there are very few formal mentorship programs available for psychiatrists starting private practice in India. Most early career psychiatrists depend on informal help from senior colleagues, teachers, or peers. There is no structured system supported by professional bodies to guide new private practitioners. Based on the participants’ responses and authors’ views, an ideal mentorship model should offer support during the early years of practice, especially for those working alone. It could include regular discussions on clinical cases, help with legal or ethical issues, and guidance on managing a clinic. Having access to senior mentors - either locally or online - would be especially helpful for those in rural or smaller towns. National psychiatric associations could play an important role in setting up such mentorship networks in the future.

The study also revealed sources of fulfilment: 44% highlighted positive patient outcomes as a key reward, and others cited professional autonomy, financial independence, and the opportunity to build trust within the community. These findings mirror international reports where mental health professionals in private practice often report greater job satisfaction when patient care aligns with personal values and autonomy is preserved [18].

The transition to private psychiatric practice appears to have a complex emotional impact on young practitioners. While a majority (57%) reported positive mental health outcomes, nearly one in five noted a negative effect, largely related to stress and burnout. This duality mirrors global findings where private practitioners value independence but also face unique emotional challenges due to professional isolation and administrative overload [19].

The study also revealed that feelings of isolation were widespread, with over 60% of respondents reporting at least occasional experiences of being unsupported. This reflects earlier findings that solo practitioners, especially in mental health, often work in environments lacking peer interaction, structured debriefing, or emotional validation [14]. Importantly, coping mechanisms relied heavily on informal social networks such as family and friends (47%) and leisure activities (34%), whereas professional resources such as therapy (6%) or peer groups (13%) were underutilized. This underuse may be due to stigma, time constraints, or lack of awareness about peer support options for mental health professionals themselves.

Engagement with professional bodies such as the IPS was high (76%), yet their perceived utility was low, with nearly 60% rating their usefulness as below average. This suggests a disconnect between membership and meaningful support, echoing previous criticisms that such bodies focus more on academic events than practical support for early career psychiatrists in private settings [10].

Stigma surrounding mental health remains a formidable barrier in psychiatric practice, especially in community-based, solo settings. In this study, over 60% of respondents rated the level of stigma in their locality as high or very high. These perceptions were reflected in clinical experiences: psychiatrists observed that stigma commonly led to delayed help-seeking, discouragement by family, and poor treatment follow-up. Such findings are consistent with national and international literature demonstrating that stigma is a key factor in underdetection, undertreatment, and disengagement from care [20,21].

The generational divide noted by 82% of respondents, where younger individuals were seen as more open to seeking psychiatric help, may suggest a gradual cultural shift. Prior studies have similarly documented that younger, urban, and more educated populations tend to hold less stigmatized views of mental illness and are more likely to seek professional help [22].

To combat community-level stigma, many respondents engaged in social media advocacy (45%), workshops (35%), and collaborations with schools or colleges (18%). This reflects a commendable move toward grassroots destigmatisation but also highlights how the burden of awareness often falls upon individual practitioners in the absence of coordinated public health initiatives. This underscores the need for institutional and policy-level interventions to address stigma at scale rather than relying solely on private efforts.

This study has several limitations that should be acknowledged. First, the use of a convenience and snowball sampling strategy may have introduced selection bias, limiting the generalizability of findings to all private psychiatrists in India. The reliance on self-report data also raises the possibility of social desirability and recall biases. Distributing the survey online through social media platforms may have introduced sampling bias, favouring psychiatrists who are more tech-savvy, urban-based, or active on digital platforms. Additionally, the cross-sectional design restricts causal inferences about the relationship between professional experiences and psychological outcomes. Furthermore, only around 2% of respondents were from rural areas, and most participants were solo practitioners. This represents a limitation in terms of generalizability, particularly for psychiatrists practicing in underserved or non-urban regions, where challenges, support systems, and patient profiles may differ significantly. Future research should aim for broader geographic coverage using stratified random sampling and consider longitudinal designs to track evolving experiences over time. Incorporating qualitative methodologies, such as in-depth interviews or focus groups, could offer richer insights into the nuanced emotional and systemic hurdles faced by early career psychiatrists. Moreover, intervention studies evaluating the impact of structured mentorship programs, digital practice tools, or institutional support systems on private practice outcomes would be valuable in guiding policy and training reforms.

## Conclusions

The findings of this study underscore an urgent need for policy-level interventions to support early career psychiatrists in private practice. Current PG training emphasizes clinical knowledge but offers limited exposure to real-world aspects of private practice, such as business management, patient communication, medico-legal literacy, and digital health integration. To bridge this gap, the National Medical Commission and relevant psychiatric bodies such as the IPS could consider integrating structured modules on entrepreneurship, ethical marketing, digital consultation platforms, and practice management into the psychiatry curriculum. Additionally, establishing formal mentorship programs, either through academic institutions or IPS chapters, could help new psychiatrists navigate early challenges more effectively. From a policy standpoint, initiatives such as subsidized professional indemnity insurance, start-up grants, or tax incentives for solo mental health practices in underserved areas could also encourage sustainable private practice models. Finally, coordinated national anti-stigma campaigns and government-endorsed mental health awareness efforts could reduce the burden that individual practitioners currently bear in public education.
